# In This Issue

**DOI:** 10.1002/cfg.331

**Published:** 2003-10


**Comparative and Functional Genomics**
*Comp Funct Genom* 2003; **4**: 459.
Published online in Wiley InterScience (www.interscience.wiley.com). **DOI:** 10.1002/cfg.331

### In this issue

### Post-normalization quality control of microarray data

John McClure and Ernst Wit applied statistical methods to the quality control of array data, enabling them to reveal such problems as mislabelling of arrays or dyes, array-wide hybridization problems, and normalization problems.

### *In silico* identification of candidate bacterial vaccine antigens

Carl Mayers *et al.* analysed the sequences of known bacterial vaccine antigens and designed an algorithm to take advantage of traits in which they differ from other proteins to allow them to predict candidate vaccine antigens from bacterial genomes.

### Proteins interacting with *Caenorhabditis elegans* G$\alpha$ subunits

Using a two-hybrid approach, Edwin Cuppen and co-workers identified several worm G$\alpha$ subunit interactors. They then used RNAi and other approaches to support their findings.

### A proposal for a standard data model for proteomics

Andrew Jones and colleagues describe their proposed standard data model for proteomics experiments, and how it could contribute to the existing community efforts in this area.

### Meeting Review: non-model fish genomics

Melody Clark and co-authors report on a recent NERC/BBSRC-sponsored workshop to discuss the resources available, and future plans, for non-model fish genomics.

### Meeting Report: genome informatics

Jo Wixon and Jennifer Ashurst report from the 2nd Genome Informatics Conference; held on the genome campus in September 2002.

### Special Section of selected reviews from the ESF Programme on Functional Genomics 1st European Conference, 'Functional Genomics and Disease 2003'

**Michael Taussig** introduces the special section, with an overview of functional genomics and disease in 2003.
**Vaclav Paces** provides a commentary on the status of genomics in the Czech Republic
**Andrea Bauer** and colleagues discuss how they have adapted array technologies in a range of ways and used them in the study of disease.
**Ulf Landegren** and colleagues describe their padlock and proximity probe methods for *in situ* and array-based DNA and protein analysis.
**Ivan Lefkovits** describes proteomic comparisons of three types of lymphocytes, resting cells, blast cells and plasma cells.
**Wagied Davids** and colleagues take us on a journey into smORFland, as they discuss the various routes by which small orphan genes might have been generated.
**Michal Linial** discusses the key criteria by which protein targets are selected for structural genomics projects and presents ProtoNet, a program that informs this process by clustering proteins using sequence and structural information.
**Jo Wixon and Joan Marsh** report on the conference, with details from a selection of the presentations from the symposia, and the plenary talks.

### Conference calendar

A listing of genomics-related conferences planned for January–March 2004.

Copyright © 2003 John Wiley & Sons, Ltd.

